# Prevalence of migraine among medical students at the University of Science and Technology, Omdurman, Sudan- A cross-sectional study

**DOI:** 10.1192/j.eurpsy.2025.1801

**Published:** 2025-08-26

**Authors:** M. A. M. Baraka, M. M. A. Adam, T. A. K. T. Al-Jabali, A. A. M. Bakheit, A. I. A. B. Abdulrhman, M. H. O. Ali, A. A. S. Ahmed, A. M. S. Hassan, A. B. A. Hag ali, F. O. I. Ahmed

**Affiliations:** 1 Faculty of medicine, University of Science and Technology, Omdurman, Sudan

## Abstract

**Introduction:**

Migraine is one of the leading neurological causes of disability worldwide with a significant impact on all aspects of life, despite the high prevalence and debilitating effects migraine remains underestimated in Sudan. This study aims to measure the prevalence of migraine among medical students at a Sudanese University.

**Objectives:**

This study aims to measure the prevalence of migraine among medical students from the University of Science and Technology Faculty of Medicine, Omdurman, Sudan.

**Methods:**

This descriptive cross-sectional study was carried out between June and August 2024 involving 283 medical students enrolled in the University of Science and Technology, data was collected online and ID-Migraine was used to screen for migraine and R programming language for statistical computation and graphics was used to analyze the data.

**Results:**

The prevalence of migraine was 41% and females were more affected than males. The most reported trigger factor was irregular sleep 84%, with rest being the most used method for relief 43% and the majority of migraine-positive students 46% mentioned their academic performance was moderately affected.

**Conclusions:**

This study found a high prevalence of migraine among medical students enrolled in the University of Science and Technology, with female students being more affected and significant impact on academic performance.

**Disclosure of Interest:**

None Declared

